# Inverse determination of the thermal contact conductance for an interface between a Co28Cr6Mo hip stem and a PMMA-based bone cement

**DOI:** 10.1038/s41598-025-89675-w

**Published:** 2025-02-13

**Authors:** Patrick Evers, Magnus Reulbach, Crystal Emonde, Henning Windhagen, Eike Jakubowitz, Sebastian Herbst, Hans Jürgen Maier, Florian Nürnberger

**Affiliations:** 1 Institut für Werkstoffkunde (Materials Science), Leibniz Universität Hannover, An der Universität 2, 30823 Garbsen, Germany; 2 Laboratory for Biomechanics and Biomaterials (LBB), Department of Orthopaedic Surgery, Hannover Medical School, Anna-von-Borries-Strasse 1-7, 30625 Hannover, Germany

**Keywords:** Thermal contact conductance, Bone cement, Co28Cr6Mo, Total hip arthroplasty revision, Induction heating, In-silico modeling, Biomedical engineering, Computational methods, Surgery

## Abstract

Traditional mechanical methods for implant and bone cement removal during total hip arthroplasty (THA) revision surgeries typically lead to surrounding tissue damage and increased risk of femoral fractures. Transcutaneous induction heating is a promising new removal approach as it causes softening of the thermoplastic bone cement, and thus prevents damage to the surrounding tissue during removal and increases stability post-revision. However, precise knowledge of the heat transfer between implant and bone cement is necessary to minimize the risk of thermal damage to surrounding tissues. In this context, knowledge of the thermal contact conductance (TCC) at the interface of Co28Cr6Mo hip stems and PMMA-based bone cement is a key issue. The present study addresses the challenge of measuring TCC by proposing an inverse method of determination using infrared thermography measurements of the heating process and a finite element simulation with a variable parameter for the TCC. Results indicate TCC values of 3,125 ± 275 Wm^− 2^K^− 1^ for dry interfaces and 5,100 ± 300 Wm^− 2^K^− 1^ for wet interfaces. The influence of heat conduction on bone cement surface temperature is significant, impacting the measured surface temperatures by 15–19% for wet and 23–30% for dry interfaces. These findings are crucial for the design of heating procedures and minimization of thermal damage during induction heating assisted THA revisions.

**Supplementary Information:**

The online version contains supplementary material available at 10.1038/s41598-025-89675-w.

## Introduction

Cemented total hip arthroplasty (THA) is a well-established and highly effective surgical procedure for managing end-stage hip arthritis and other severe hip pathologies. This technique involves the fixation of prosthetic components to the bone using polymethylmethacrylate (PMMA)-based bone cement and has demonstrated remarkable long-term clinical outcomes. The success of cemented THA is largely attributed to the stable and immediate fixation it provides, allowing for early weight-bearing and mobilization of patients post-operatively. Data from the German Implant Registry suggest an 8-year implant survival rate of 95.9% for cemented femoral components, indicating the durability and reliability of cemented THA, particularly in elderly populations who may not have the bone quality required for cementless fixation^1^.

Moreover, the advances in cementing techniques have significantly improved the longevity of cemented THA. Modern techniques emphasize thorough canal preparation, pulsatile lavage, and the use of a cement gun to ensure optimal cement penetration and interlocking with the cancellous bone^2^. However, PMMA-based bone cement is biologically inert and does not chemically bond with the surrounding bony tissue. This allows for micromotions during regular activities causing abrasive wear of the cement mantle. This abrasion creates wear particles at the bone-cement interface that can lead to osteolysis, and thus aseptic loosening^3^. Aseptic loosening, together with infections and periprosthetic fractures, remains among the main causes for revision surgery in THAs^1,4^.

The treatment of these complications requires revision surgery, during which the implant is removed and the bone is prepared for the reimplantation of a femoral stem. Conventional revision methods use mechanical means both for the dislocation of the implant and for the removal of the remaining bone cement. The use of slide hammers on the implant and of chisels and grinding burrs on the bone cement oftentimes cause damage of the surrounding tissue. Thus, THA revisions lead to femoral fractures in up to 50% of cases^5,6^. This not only increases healing times but also significantly increases the risk of re-revision^1^.

More modern methods such as laser or ultrasonic removal are currently under investigation as more gentle removal procedures but generally prolong surgeries due to their low rates of material removal^7,8^. A promising new approach is the use of induction heating technology for the transcutaneous heating of the implant at its interface with the bone cement^9,10^. The heat generated leads to a softening of the bone cement and facilitates its and the implant’s removal^11^. Thus, more of the surrounding tissue can be preserved leading to shorter convalescence periods and improved stability of the revision endoprosthesis. However, precise knowledge of heat transfer is necessary to predict the temperature distribution in the bone cement upon heating. This is especially critical to ensure thermal damage to the surrounding tissues is kept to a minimum. Due to the novelty of the procedure, no sources for the thermal contact conductance of PMMA-based bone cement and Co28Cr6Mo alloys are available in literature. Thus, measurements of said contact conductance are necessary.

However, the thermal contact conductance at the interface between bone cement and implant cannot be measured directly since the disc specimens that are usually employed for the measurement of heat conductivity can only be manufactured for each material individually. In preliminary tests, the interface between cement layers and disc-shaped specimens of CoCrMo split during or after curing of the cement. PMMA-based bone cements and Co28Cr6Mo alloys, which are most commonly used for cemented THAs, have dissimilar heat expansion coefficients and the polymerization of the bone cement generates heat, leading to temperatures between 70 °C and 115 °C^12,13^. The splitting of layered circular specimens was attributed to the difference in thermal expansion during cooling of specimens. Additionally, bone cement features no adhesive properties relevant for the interface with Co28Cr6Mo. A different approach of measuring the heat conduction coefficient while applying external pressure to the interface necessitates the knowledge of the pressure on the contact area between Co28Cr6Mo and bone cement in-situ. These data are not readily available in literature and can only be determined with great effort. Thus, in the present study a method of inverse determination of thermal contact conductances for specimens with a geometry similar to that of bone cement layers on the stem of a hip implant using in-silico modeling and fitting to experimental data is proposed.

## Methods

### Experimental

Eight cylindrical specimens with an outer diameter of 13 ± 0.02 mm and a length of 100 mm were manufactured from a Co28Cr6Mo alloy (DIN EN ISO 5832-12) by turning. The material composition is given in Supplementary Table S1^14^.

The mantle surfaces of the metallic specimens were characterized using a confocal laser microscope (Keyence Corporation, Osaka, Japan) and an average surface roughness of *R*_a_ = 1.54 μm was measured, which is similar to that of commercially used implants^15^. A layer of Palacos R bone cement (Heraeus Medical GmbH, Wehrheim, Deutschland) was deposited onto two thirds of the lateral surface of each specimen using a PALAMIX vacuum mixing system (Heraeus Medical GmbH, Wehrheim, Deutschland) and after curing turned down to a thickness of 1 ± 0.04 mm. To achieve a homogenous and even distribution of the bone cement, a mold was milled from Polytetrafluorethylene (PTFE), holding a tube made of polylactic acid (PLA) which was filled with bone cement into which the metallic specimens were then inserted. This closely resembles the procedure of implant placement during cemented THAs^2^. The bone cement layer was cured for 1 h inside the mold before the PMMA-coated Co28Cr6Mo specimens were removed. Supplementary Figure S2 shows the specimens before coating, the setup used for the attachment of the bone cement and the specimen after turning of the cement mantle to dimension.

Four of the specimens were stored in air at 37 °C for 14 days, while the other four specimens were submerged in Ringer’s solution at 37 °C for 14 days according to the procedure described by Reulbach et al.^11^. After completion of the storage period, the specimens were inductively heated using an induction heating test bench with an MFG30 induction generator (eldec Induction GmbH, Dornstetten, Deutschland). In this test bench, the cylinders were fixed longitudinally in the center of a five-turn inductor with an inside diameter of 25.4 mm and a total length of 40 mm so that the end of the specimen lined up with the end of the inductor as shown in Supplementary Figure S3.

The target value for apparent power of the induction generator control was set to 3.9 kW for a heating duration of 1 s and resulting currents inside the inductor were monitored using a current sensing coil MA20 (Chauvin Arnoux Metrix, Asnières-Sur-Seine, Frankreich) connected to a high frequency oscilloscope Waverunner Zi640 (Teledyne LeCroy, Chestnut Ridge, USA). From the signals recorded, average values of current and frequency were determined using Fast Fourier Transformation in MATLAB R2024a (The Mathworks Inc., Natick, USA).

During heating, the surface temperature of the bone cement was recorded using a thermography camera PI450 (Optris GmbH, Berlin, Germany) with a sampling rate of 80 Hz and a measurement accuracy of ± 2 °C. The setup used is depicted in Supplementary Figure S4.

The emission coefficient of the bone cement was determined in comparative measurements with thermocouples on oven-heated specimens to be ε = 0.92. For each specimen, the maximum surface temperature was ascertained and time-temperature-data for the corresponding position on the cement mantle were used as a reference for modeling. An example output frame of the thermal imaging system is shown in Supplementary Figure S5.

### Modeling

A model in the finite element method simulation software Ansys 23.1 (Ansys Inc., Canonsburg, USA) was used for the in-silico simulation of heat generation and transfer. The model developed consists of a harmonic subtask for the induction heating of the metallic specimen, in which the current is applied sinusoidally, and a transient thermal subtask for the heat transfer, in which the temperature distribution is calculated over time.

In the electromagnetic subtask, Maxwell’s Eqs. (1)–(4) are solved where *dl*, *da* and *dv* are the smallest differential forms of a line, an area and a volume respectively.

1 $$\:{\oint\:}_{line}^{}E\:dl=\:-\frac{d}{dt}{\oint\:}_{surface}^{}B\:da$$

2 $$\:{\oint\:}_{line}^{}H\:dl=\:\frac{d}{dt}{\oint\:}_{surface}^{}D\:da+{\oint\:}_{surface}^{}J\:da$$

3 $$\:{\oint\:}_{surface}^{}E\:da=-\frac{d}{dt}{\oint\:}_{volume}^{}\:\gamma\:\:dv$$

4 $$\:{\oint\:}_{surface}^{}B\:da=0$$

*E* is the electric field, *B* the magnetic flux density, *H* the magnetic field, *D* the flux density, *γ* the electric charge density and *J* the current density.

The thermal task solves the first law of thermodynamics given in Eq. (5) where *Ρ* is the density, *ρ* the electrical resistivity, *q*_F_ the surface heat flux enclosing the domain, *T* the temperature, *C* the specific heat capacity and *Q*_em_ the electromagnetically generated heat relating to the current density calculated in the electromagnetic subtask as per Eq. (6).

5 $$\:\frac{d}{dt}{\oint\:}_{volume}^{}\:{\rm\:P}CT\:dv=-{\oint\:}_{surface}^{}{q}_{\text{F}}\:da+{\oint\:}_{volume}^{}\:{Q}_{\text{e}\text{m}}\:dv$$

6 $$\:{Q}_{\text{e}\text{m}}=\rho\:{J}^{2}$$

**Fig. 1:**
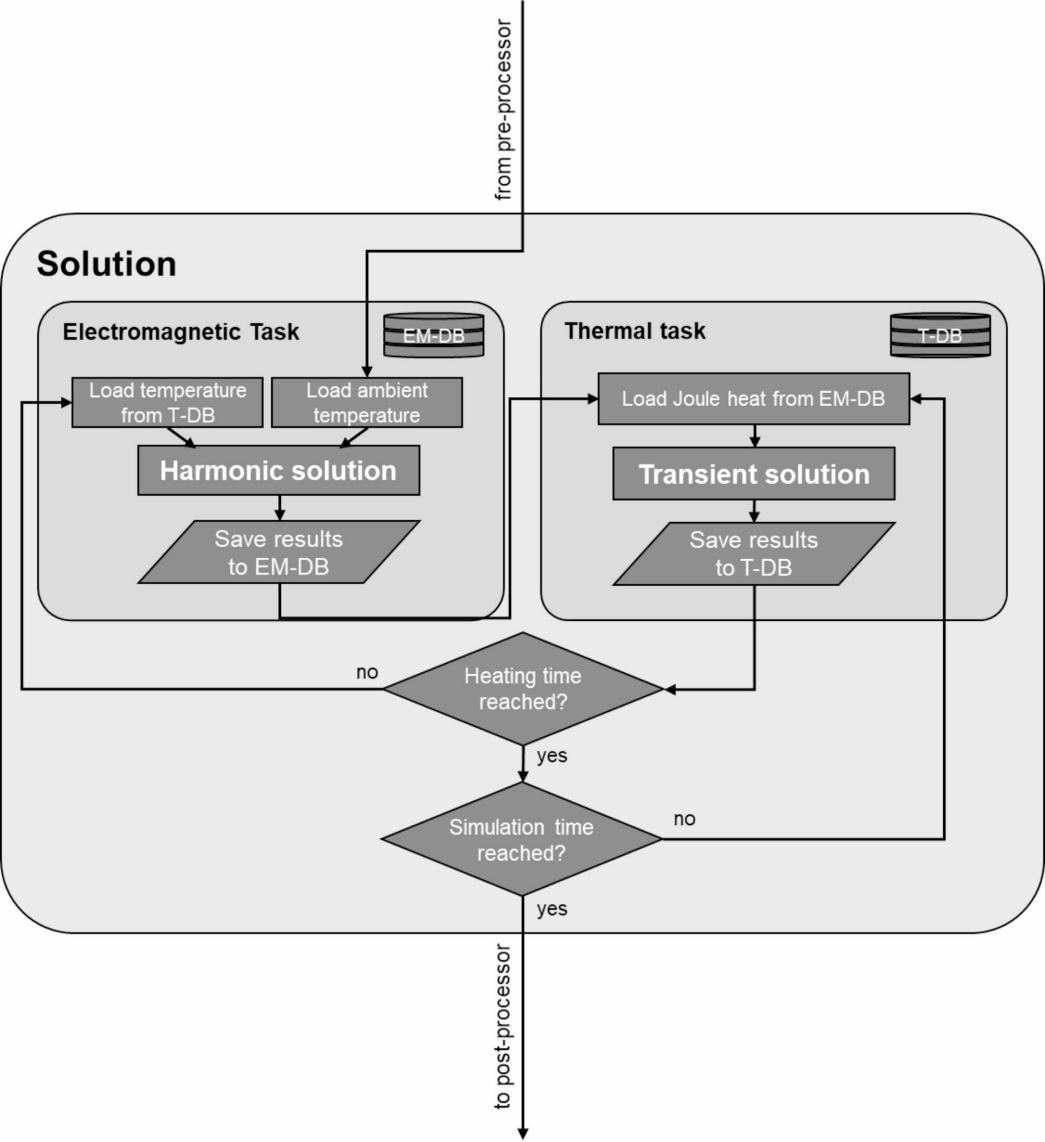
Simulation program flow chart. EM-DB is the database of electromagnetic results. T-DB is the database of thermal results.

The solution of both subtasks was alternated for every 0.1 s time interval. The model is based on the one described in^14^. In addition to the model described there, a bone cement layer was added to the transient thermal task and a variable heat conduction coefficient for the interface of Co28Cr6Mo and bone cement was introduced. Figure 1 shows a flow chart of the simulation program with the two consecutively repeated subtasks in the solution step.

**Fig. 2:**
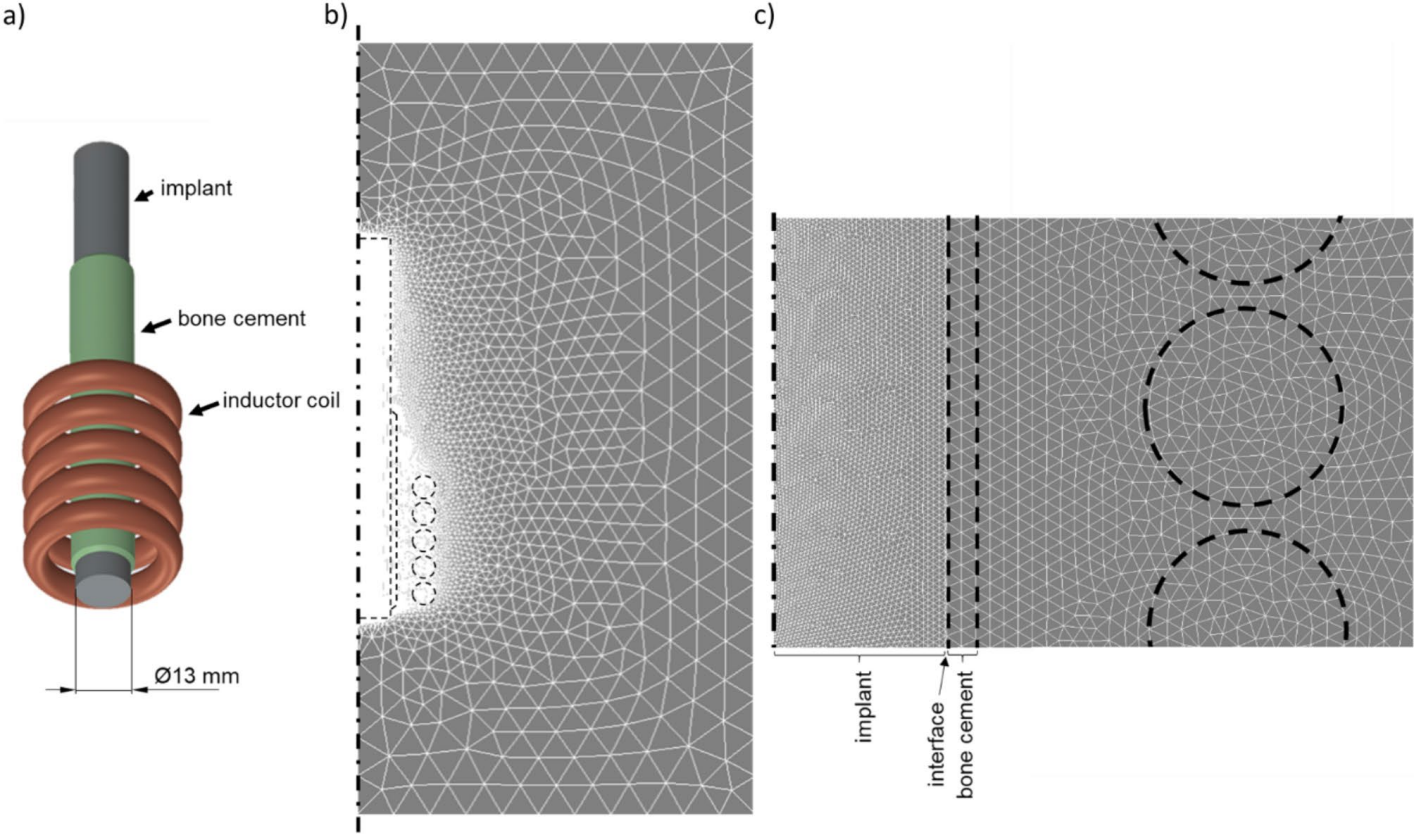
Geometry used in the simulation (**a**) and cross section of the mesh (**b**) with enlarged cutout (**c**).

The induction coil was approximated as individual inductor rings that were each loaded with the inductor current and frequency. Other than that, the specimen and coil dimensions were identical to those described in the experimental section. The actual geometry used for the simulations is shown in Fig. 2. To reduce computational resources required for calculations, the axial symmetry of the model was used and a 5-degree slice along the longitudinal axis was simulated.

For meshing of the individual components in the model, different maximum edge lengths were defined as multiples of the skin depth calculated for Co28Cr6Mo – in this case 3.42 mm at 17.8 kHz. The meshing factors used in the calculations and the resulting maximum edge lengths for the average frequency observed in the experiments are given in Supplementary Table S6. It was ensured that no element shape checking warnings were produced and convergence was established for the maximum edge lengths selected. The coil, implant and cement volumes were meshed with elements of type SOLID236 for the electromagnetic task. SOLID236 is a three-dimensional element with 20 nodes capable of modelling electromagnetic fields. For the thermal task, the air and coil volumes were defined as null elements, while the implant and bone cement volumes were redefined as elements of types SOLID279. This element type is a higher order three-dimensional element with a temperature degree of freedom at each node. Additionally, the thermal contact between implant and bone cement was modelled with CONTA174 elements on the implant surface and the corresponding TARGE170 elements on the inner surface of the bone cement.

Thermal diffusivities of Co28Cr6Mo and Palacos R bone cement were determined using an LFA 447 NanoFlash Analyzer (Erich NETZSCH B.V. & Co. Holding KG, Selb, Germany). The density of dry bone cement and Co28Cr6Mo were measured using an MK2200 density measuring scale (MK Industrievertretungen, Stahlhofen am Wiesensee, Germany). The density of wet bone cement was calculated in accordance with^11^. Specific heat capacities of dry bone cement and Co28Cr6Mo were measured by differential scanning calorimetry using a DSC 214 Polyma (Erich NETZSCH B.V. & Co. Holding KG, Selb, Germany), while the specific heat capacity of wet bone cement was calculated in accordance with^11^. The magnetic permeability of the Co28Cr6Mo alloy was verified using a Magnetoscop 1.070 (Institut Dr. Foerster GmbH & Co. KG, Reutlingen, Germany). Material and input parameters of the model that were assumed as temperature-independent for the expected temperatures are given in Supplementary Table S7. The input parameters of the model that included temperature dependence are given in Supplementary Table S8.

For each calculation, the thermal conduction coefficient *h*_c_ between the bone cement and the Co28Cr6Mo cylinder was varied between 1,000 and 10,000 Wm^− 2^ K^− 1^ in steps of 50 Wm^− 2^K^− 1^. All other parameters were kept identical for all calculations. Time-temperature curves for the position where the maximum surface temperature was measured in the experiments were calculated for a simulation time of 10 s including 1 s of heating. The calculated temperatures were then compared with temperatures measured in the experiments by determining the mean square error (MSE), the difference in average temperature increase rate and the maximum deviation inside a time segment from 1.5 s to 4.5 s. During this period, surface temperatures increase significantly and convection losses play an insignificant role, and thus were neglected in the calculations. Values determined for *h*_c_ were deemed acceptable if they produced an absolute maximum deviation of less than 2 K, a maximum error of ± 0.68 K/s for the temperature increase rate and a maximum MSE of ± 0.3 K.

## Results

A comparison between the measured temperatures and the calculated temperatures is shown in Supplementary Figure S9 to illustrate the effects of a significantly too low, a significantly too high and a suitable value for *h*_c_. A value of 100 Wm^− 2^K^− 1^ was chosen to illustrate the effects of underestimated thermal contact conductance, a value of 3,300 Wm^− 2^K^− 1^ was chosen as an example of a suitable fit and a value of 10^8^ Wm^− 2^K^− 1^ was chosen to show the simulation results for close to ideal thermal contact conductance.

Table 1 shows the frequency and currents measured during experiments for the dry and wet specimens.

**Table 1:** Inductor frequencies and currents measured during experiments for dry and wet specimens.

					Mean	Standard deviation
Experiment ID	1	2	3	4		
Storage condition	Dry	Dry	Dry	Dry		
Frequency in Hz	17,778	17,784	17,783	17,784	17,782	2
Current in A	1,009	1,030	1,030	1,010	1,020	10
Experiment ID	5	6	7	8		
Storage condition	Wet	Wet	Wet	Wet		
Frequency in Hz	17,786	17,772	17,782	17,788	17,782	6
Current in A	1,021	1,000	1,030	1,029	1,020	12

**Fig. 3:**
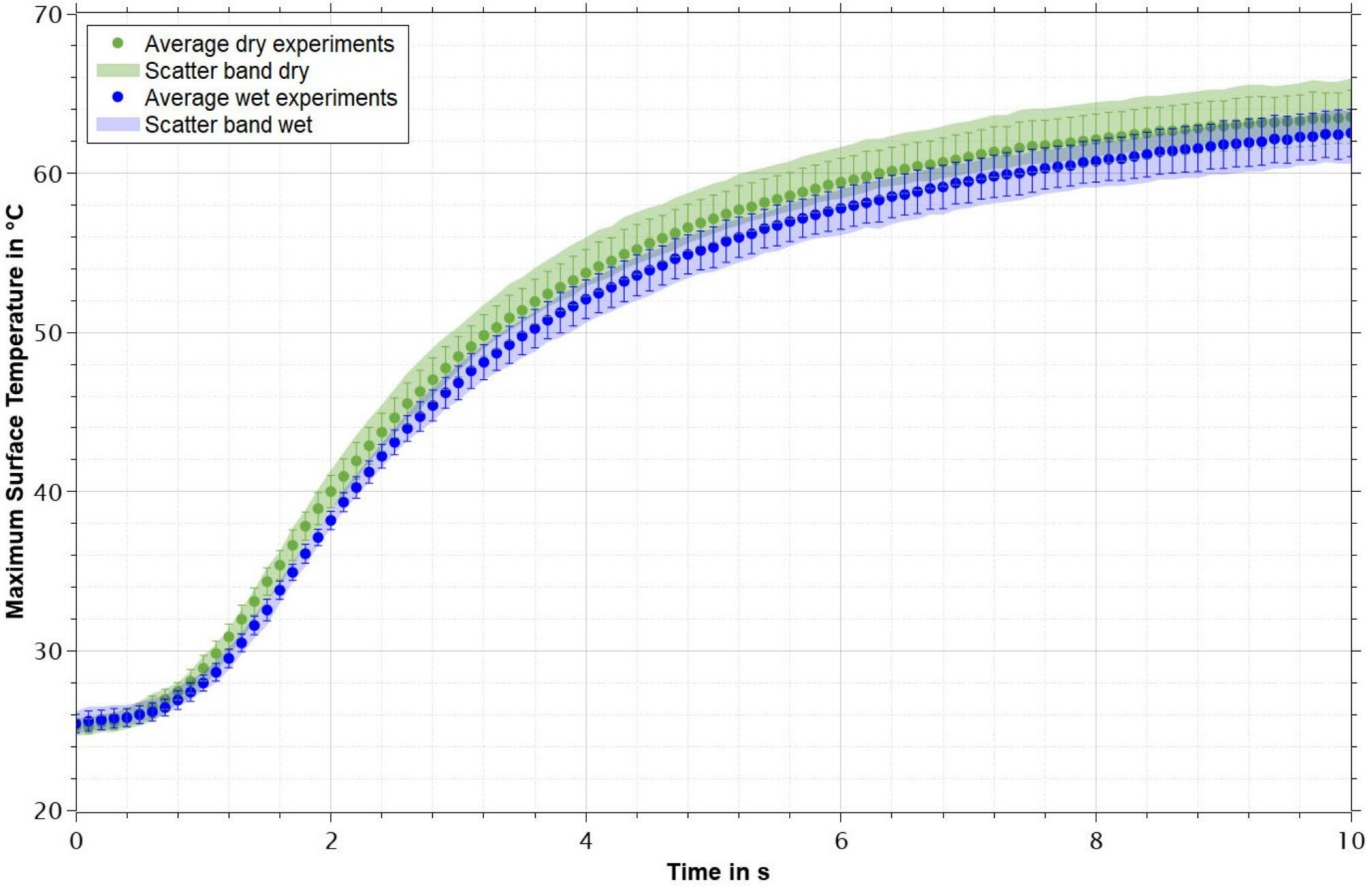
Averaged maximum surface temperature for dry and wet specimens with standard deviations and respective scatter bands for all samples.

The averaged maximum surface temperatures measured for the four dry and four wet specimens with standard deviations and scatter bands for dry and wet specimens respectively is shown in Fig. 3. It can be seen that maximum surface temperatures of dry specimens exceed those of wet samples in almost all cases, while all values are tightly grouped together, reaching temperatures of 62 °C (dry specimens) and 61 °C (wet specimens) after 10 s of measurement on average. Both averages as well as both scatter bands show a distinct delay in the rise of temperatures between 0 and 0.8 s, a sharp increase between 0.8 and 2.5 s and a consecutively slowing rise in temperature.

The experimentally determined time-temperature curves were then compared against the calculated time-temperature curves for each experiment. Using the following figures as examples for one dry and one wet specimen, the error metrics used in the evaluation of simulation results and the procedure of determining acceptable and optimal ranges for TCC are illustrated.

Supplementary Figure S10 shows the analysis of maximum deviation, slope error and MSE for values of *h*_c_ varying from 2,300 Wm^− 2^K^− 1^ to 4,900 Wm^− 2^K^− 1^ for specimen 2 that was stored in dry conditions. An acceptable range was determined as including all values for *h*_c_ where the error criteria previously defined were met. For this specimen the acceptable range was determined to be 3,200 Wm^− 2^K^− 1^ < *h*_c_ < 3,700 Wm^− 2^K^− 1^ and the best fit was reached at *h*_c_ = 3,300 Wm^− 2^K^− 1^. For determining optimal simulation quality, the value of *h*_c_ for which the sum of MSE and maximum deviation is minimal was chosen and minimal slope error was used in case of equality.

The calculated time-temperature profile when employing the optimal value of *h*_c_ (3,300 W m^− 2^ K^− 1^) in comparison to the measured temperature of the experiment with specimen 2 is shown in Supplementary Figure S11.

The analysis of maximum deviation, slope error and MSE for values of *h*_c_ varying from 3,600 Wm^− 2^K^− 1^ to 6,000^− 2^ K^− 1^ for specimen 7 that was stored in wet conditions is depicted in Supplementary Figure S12. For this specimen the acceptable range was determined to be 4,200 Wm^− 2^K^− 1^ < *h*_c_ < 6,000^− 2^ K^− 1^ and the best fit was reached at *h*_c_ = 5,300 Wm^− 2^K^− 1^. In general, maximum deviation, slope error and MSE are on a lower level (absolute values) compared to the dry bone cement. This was the case for all specimens stored in wet conditions.

The resulting simulated time-temperature profile for the optimal value of *h*_c_ in comparison to the maximum surface temperature determined in the experiment of specimen 7 with error indicators showing the precision of the thermography camera used can be seen in Supplementary Figure S13.

These analyses were conducted on all eight specimens and the average of the optimal values of *h*_c_ were determined for dry and wet specimens. The results are given in Table 2 for dry specimens and Table 3 for wet specimens.

**Table 2:** Maximum surface temperatures after 10 s of the experiment, minimum, maximum and optimal values for *h*_c_ as determined from simulation and averages for these values for dry specimens.

	Experiment ID				Mean	Standard deviation
	1	2	3	4		
Storage condition	Dry	Dry	Dry	Dry		
Maximum surface temperature at 10 s in °C	62.4	62.4	65.9	63.4	63.5	1.4
Minimum acceptable *h*_c _in W m^−^² K^− 1^	3,200	3,200	2,550	2,500	2,863	338.0
Maximum acceptable *h*_c _in W m^−^² K^− 1^	3,500	3,700	2,850	2,900	3,238	369.8
Optimal *h*_c _in W m^−^² K^− 1^	3,400	3,300	2,850	2,900	3,113	240.8

**Table 3:** Maximum surface temperatures after 10 s of the experiment, minimum, maximum and optimal values for *h*_c_ as determined from simulation and averages for these values for wet specimens.

	Experiment ID				Mean	Standard deviation
	5	6	7	8		
Storage condition	Wet	Wet	Wet	Wet		
Maximum surface temperature at 10 s in °C	63.6	62.1	63.8	60.6	62.5	1.3
Minimum acceptable *h*_c _in W m^−^² K^− 1^	4,650	4,100	5,000	5,250	4,750	431.6
Maximum acceptable *h*_c _in W m^−^² K^− 1^	5,500	4,800	5,700	5,400	5,350	335.4
Optimal *h*_c _in W m^−^² K^− 1^	5,100	4,800	5,300	5,400	5,150	229.1

From these values, the identified optimal range for dry contacts of PMMA-based bone cement and Co28Cr6Mo was determined to be 2,850 Wm^− 2^K^− 1^ < *h*_c_ < 3400 Wm^− 2^K^− 1^. The identified optimal range for wet contacts of PMMA-based bone cement and Co28Cr6Mo was determined to be 4,800 Wm^− 2^K^− 1^ < *h*_c_ < 5,400 Wm^− 2^K^− 1^.

**Fig. 4:**
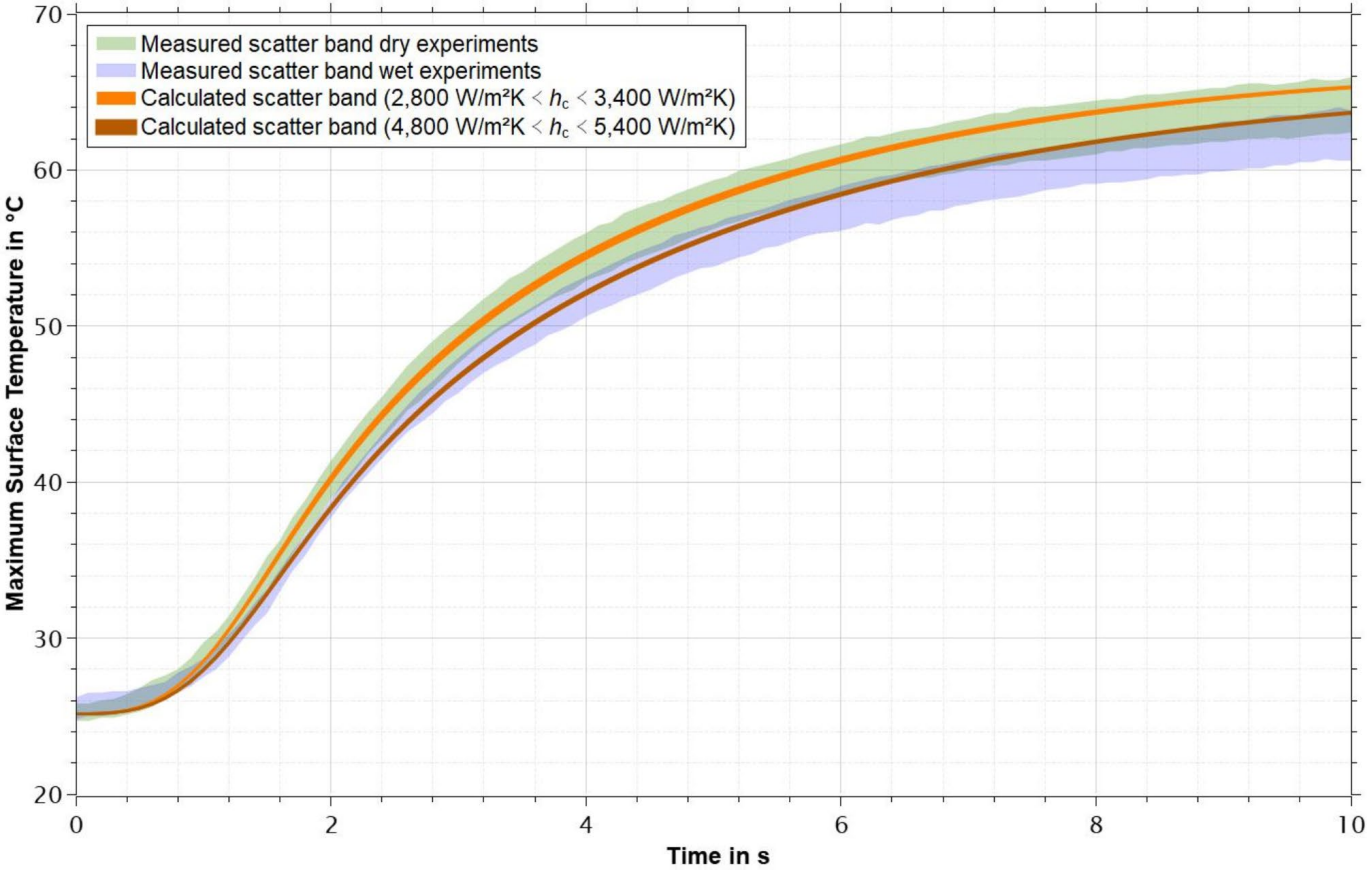
Calculated maximum surface temperatures resulting from the identified optimal values of *h*_c_ for dry and wet specimens respectively in comparison to scatter bands of maximum surface temperatures determined experimentally.

The simulated time-temperature data for dry and wet specimens in comparison to the scatter bands previously shown are given in Fig. 4. The light and dark orange bands show the range of possible calculated temperatures resulting from all values of *h*_c_ in the optimal range for dry and wet specimens respectively. It can be seen that all values of *h*_c_ determined as optimal produce a suitable prediction of temperatures for the respective specimen conditions both in shape of the curve and magnitude of the temperatures reached.

## Discussion

The test bed allowed reproducible positioning of the samples inside the inductor, which lead to a high reproducibility of inductor frequencies and currents for all experiments. No significant difference in either frequency or current was observed between dry and wet specimens which was expected, given that the effect of water on the electromagnetic field is negligible in comparison to the effects of Co28Cr6Mo.

Wet specimens show values around 2,000 Wm^− 2^ K^− 1^ higher than dry samples both individually and on average. This can be explained by the relatively small area of bone cement being in contact with the Co28Cr6Mo on a microscopic scale due to roughness and surface imperfections of both materials. Air filling the gaps in the contact plane is a comparatively bad conductor for heat thus hampering heat flow into the bone cement. In wet specimens this air is at least partially replaced by water which can both adhere to the surfaces better and is a better heat conductor, thus increasing thermal contact conductance. Both the consistency in increase of thermal contact conductance between wet and dry specimens and the consistency of absolute values for *h*_c_ amongst specimens of the same condition demonstrate a good repeatability in the production of the contact between PMMA and Co28Cr6Mo in all samples.

However, heating of wet specimens did not lead to increased maximum surface temperatures in the experiments of the present study, most likely because the higher specific heat capacity counteracted the larger thermal contact conductance for the bone cement layer thicknesses investigated. The effects observed with wet specimens are beneficial for the planned application in induction heating, as water facilitates heat transfer to the bone cement and acts as a thermal buffer protecting surrounding tissues from excessive heat.

Although surface roughness was neglected as an influencing factor, it is expected that the ranges of values determined for *h*_c_ will be applicable to most common endoprostheses with blasted surfaces, since the specimens used deliberately featured an average surface roughness typical for cemented hip endoprostheses. Especially for wet specimens the influence of surface finish is expected to be compensated for by water filling the gaps in the interface layer.

Data for other polymers in contact with metallic surfaces can be used as a reference for evaluating the plausibility of the values determined for *h*_c_. As injection molding of thermoplastics resembles the process of implant insertion into paste-like bone cement, data gathered for the interface of thermoplastics and mold surface can be used. Here, values of 1,250 Wm^− 2^K^− 1^ and 4,000^− 2^ K^− 1^ can be found for a contact of steel and polypropylene^16^. For a contact of PMMA and copper-beryllium alloy Nakao et al. measured a thermal contact resistance of 0.00037 m^2^KW^− 1^, equating to a thermal contact conductance of 2,700 Wm^− 2^K^− 1^^17^. Zhou at al. observed thermal contact conductances of 2,700 Wm^− 2^K^− 1^ to 6,000^− 2^ K^− 1^ for polyamide/polyamide contact at 72 °C^18^. For cyclic olefin copolymers in contact with steel molds Su et al. measured heat transfer coefficients of up to 9,000^− 2^ K^− 1^ for 40 MPa of packing pressure^19^. Dawson et al. characterized a PMMA-steel interface and found a value of 2,500 Wm^− 2^K^− 1^ for an airgap of 0.02 mm, which represents a pressure-free contact. Given that the specimens in the present study should be free of any macroscopic air gaps due to the manufacturing procedure, the value given by Dawson et al. can be considered a minimum for the expected thermal conductance coefficients. Although the bone cement investigated in the present study contains around 10% zirconium dioxide as an X-ray contrast agent^20^ and is thus not identical to the pure polymer samples used in literature, the values determined are within the range given in previous publications. A consideration of the proportion of temperature difference caused by the thermal contact conductance to the overall temperature difference of a specimen can be used to assess the influence of the TCC values determined in the present study on the total heat transfer. For this, the influence of thermal contact conductance at the interface on bone cement surface temperature can be expressed as the following quotient *δ*_TCC_

7 $$\:{\delta\:}_{\text{T}\text{C}\text{C}}=\:\frac{{T}_{\text{s}\text{u}\text{r}\text{f}\text{a}\text{c}\text{e},\:\text{m}\text{e}\text{t}\text{a}\text{l}}-{T}_{\text{i}\text{n}\text{t}\text{e}\text{r}\text{f}\text{a}\text{c}\text{e},\:\:\text{b}\text{o}\text{n}\text{e}\:\text{c}\text{e}\text{m}\text{e}\text{n}\text{t}}}{{T}_{\text{s}\text{u}\text{r}\text{f}\text{a}\text{c}\text{e},\:\text{m}\text{e}\text{t}\text{a}\text{l}}-{T}_{\text{s}\text{u}\text{r}\text{f}\text{a}\text{c}\text{e},\:\:\text{b}\text{o}\text{n}\text{e}\:\text{c}\text{e}\text{m}\text{e}\text{n}\text{t}}}$$

Since both the temperature of the metal component and the temperature of the bone cement are the control variables in the subsequent induction heating application, temperatures are used instead of heat, deliberately neglecting the influence of the different heat capacities.

**Fig. 5:**
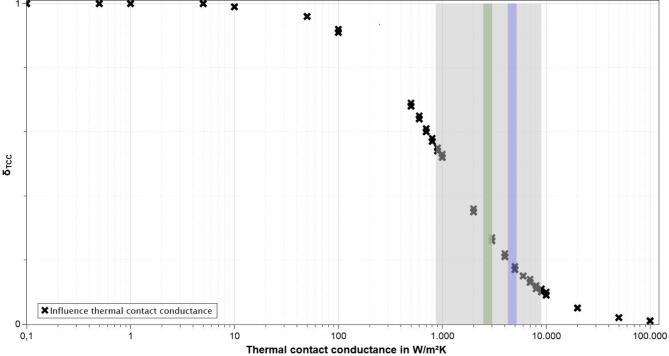
Comparison of the proportion of surface temperature difference caused in relation to the thermal contact conductance (logarithmic scale) at the interface; the grey area shows the range of values found in literature, the green and blue areas represent the values identified in the present study for dry and for wet specimens respectively.

The proportion of temperature difference between the maximum temperature at the metallic surface and the maximum temperature at the bone cement surface generated by thermal contact conductance and thermal conduction within the bone cement respectively is depicted in Fig. 5. For this, simulations were carried out for various thermal contact conductances between 0.1 and 100,000 Wm^− 2^ K^− 1^ for specimen surface temperatures ranging from 20 °C to 500 °C. The close grouping of data points shows the negligible influence of absolute surface temperature. This was expected from the fact that the relevant material parameters change only slightly with temperature. The grey area indicated the values commonly found in literature, while the green and blue areas correlate with the values determined in the present study.

It can be seen that thermal contact conductance is responsible for between 15 and 19 % of temperature difference for wet bone cements and between 23 % and 30 % of temperature difference for dry bone cements. Therefore, TCC is an important influencing factor for the achievable surface temperatures of indirectly induction-heated bone cements and must be considered in in-silico modeling. Knowledge of this parameter is crucial especially in areas of thinly applied bone cements.

Uncertainties in the model parameters obtained from literature, deviations in the positioning of the specimens in the coil and slight variations in the composition of the bone cement due to manual mixing could negatively influence the generalizability of the results obtained. Additionally, the measurement uncertainty of the thermography camera could have negatively influenced the accuracy of the experiments. However, the thermal contact conductances were determined to a significantly higher degree of accuracy than previously available in literature. Additionally, a bone cement thickness of more than 2 mm is expected in the clinical application which further lessens the influence of said uncertainties. It can be expected that uncertainties introduced in positioning of the implant during primary surgery and from application of heating during revision surgery will have a greater influence on bone cement surface temperature than uncertainties of the thermal contact conductances determined.

The resulting difference in temperature distribution in the cross section of the sample geometry for the average dry specimen is illustrated in Supplementary Figure S14. The knowledge of thermal contact conductance allows for a more precise modeling of induction heating of cement-coated implants in the future, enabling improved coil design and the definition of heating strategies with minimal thermal damage to the surrounding tissues. By extending the model with the addition of a bone layer and thermal contact conductance at the bone cement-bone-interface, predictions on the thermal load of the bone will become possible. Additionally, a damage parameter could be calculated from this information to predict histological damage of the femur during induction heating-assisted implant removal.

This information will allow for the prediction of temperature distributions during induction heating in revision arthroplasty surgeries. The knowledge of heat generation and transfer in cemented paramagnetic hip implants enables the design of inductors and heating regimes that can be used by surgeons during revision surgery to create a localized hyperthermia in the implant leading to decreased stability of the bone cement mantle and subsequently to a facilitated removal.

## Conclusions

IR thermography and an ANSYS in-silico model of inductive heating were successfully used for the inverse determination of thermal contact conductances at the interface of Co28Cr6Mo alloy and PMMA-based bone cement. The main results can be summarized as follows:

1. For dry interfaces the values for *h*_c_ were determined to be in the range of 3,125 ± 275 Wm^-2^K^-1^, and for wet interfaces the *h*_c_ values were determined to be 5,100 ± 300 Wm^-2^K^-1^.
2. The increased specific heat capacity of wet bone cements counteracted the effect of increased thermal contact conductance resulting in temperatures slightly lower than those determined for dry specimens.
3. The influence of heat conduction at the interface on the outside bone cement temperature is significant, ranging between 15 % and 19 % for wet bone cements and between 23 % and 30 % for dry bone cements.

## Electronic supplementary material

Below is the link to the electronic supplementary material.

Supplementary Material 1

## Data Availability

The datasets used and/or analysed during the current study are available from the corresponding author upon reasonable request.
