# Supplementary material for: Inverse determination of the thermal contact conductance for an interface between a Co28Cr6Mo hip stem and a PMMA-based bone cement
Source: Sci Rep. 2025 Feb 13;15:5328. doi: 10.1038/s41598-025-89675-w (PMC11825923; doi:10.1038/s41598-025-89675-w)
Supplement: Supplementary file 1 — Supplementary Material 1 [file 41598_2025_89675_MOESM1_ESM.pdf]

Supplementary Table S1. Chemical composition of the Co28Cr6Mo alloy used in the experiments as determined by EDS measurements [1]

wt. %	***Co***	***Cr***	***Mo***	***Mn***	***Fe***	***Ni***
	63.3	29.3	6.0	0.6	0.3	0.5

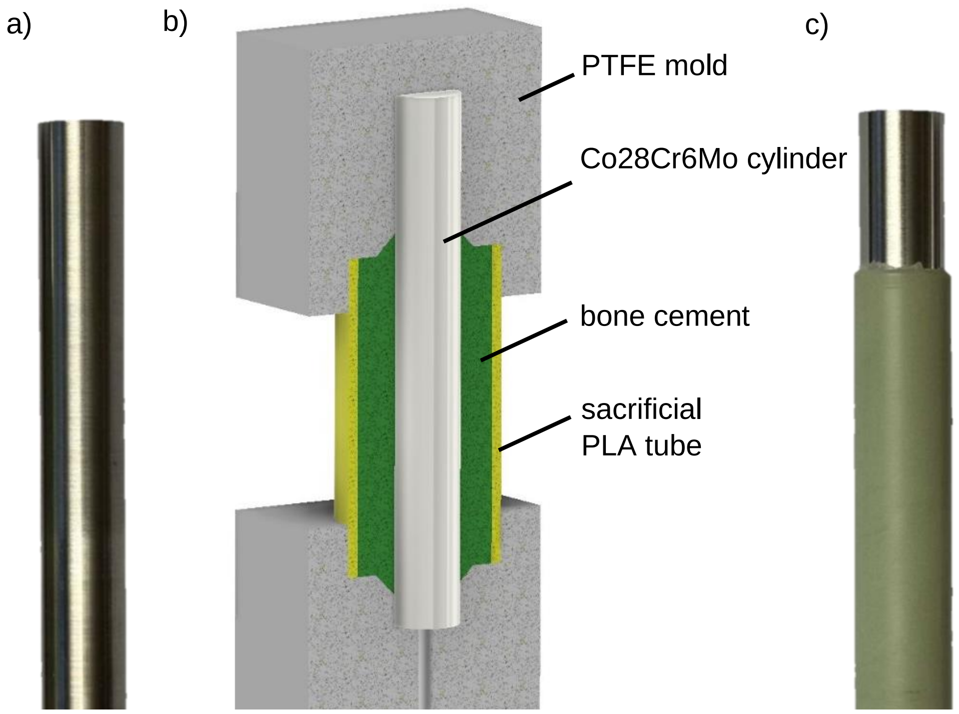

Supplementary Figure S2 Steps in manufacturing of the specimens: a) uncoated Co28Cr6Mo cylinder, b) schematic of cylinder in the mold, c) specimen after turning of bone cement layer to dimension

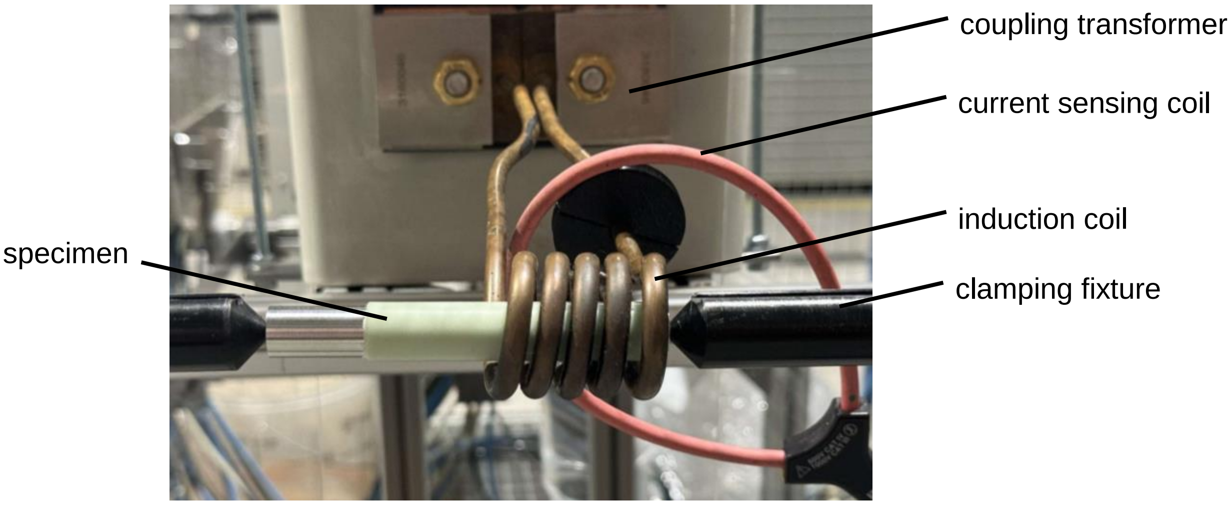

Supplementary Figure S3 PMMA-coated Co28Cr6Mo specimen inside the induction coil; see main text for details

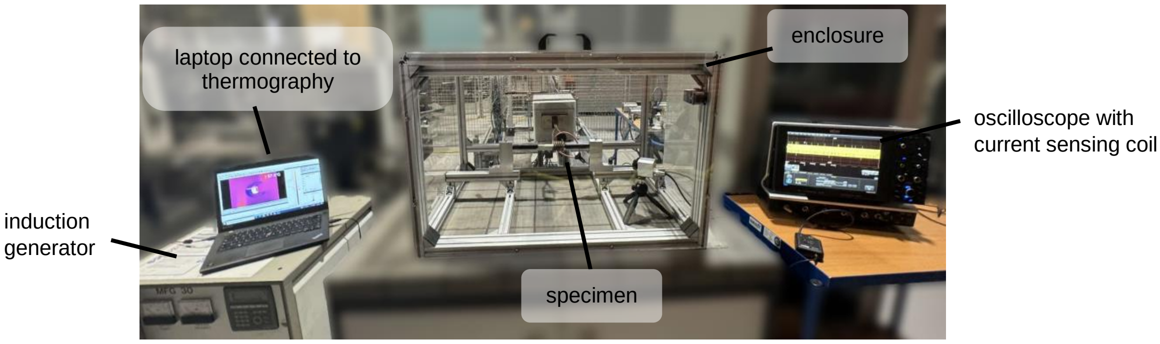

Supplementary Figure S4 Test setup used for the induction heating experiments

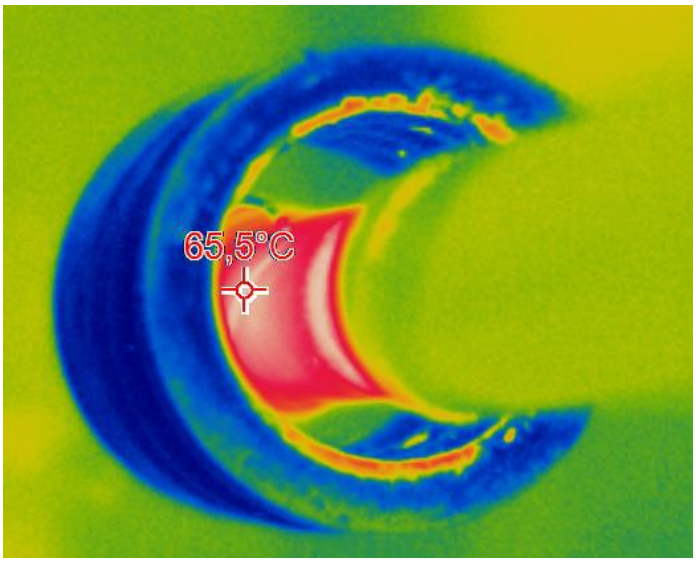

Supplementary Figure S5 False color image produced by the thermography camera for specimen 4 after 9.8 s of measurement; the hotspot indicated by the cross-hair symbol at a temperature of 65.5 °C

Supplementary Table S6 Meshing parameters per component used in the in-silico model

component	meshing factor	resulting maximum edge length
**coil**	0.5	1.71 mm
**free space inside coil**	0.5	1.71 mm
**free space outside coil**	2	6.83 mm
**bone cement interface**	0.2	0.68 mm
**metallic specimen center**	1	3.42 mm
**metalllic specimen interface**	0.2	0.68 mm

Supplementary Table S7 Input parameters of the model, which were considered to be temperature-independent for the relevant temperature range. Densities are averaged from four specimens.

variable	symbol	unit	value	reference
magnetic permeability Co28Cr6Mo	$\mu_{Co28Cr6Mo}$	H/m	1.001	[2]
electrical resistivity Co28Cr6Mo	$R_{Co28Cr6Mo}$	Ωm	$8.21 \cdot 10^{-7}$	[3]
magnetic permeability Cu	μ_{Cu}	H/m	$1 - 6.4 \cdot 10^{-6}$	[4]
electrical resistivity Cu	R_{cu}	Ωm	$1.78 \cdot 10^{-8}$	[4]
magnetic permeability air	μ_{air}	H/m	1	
magnetic permeability PMMA	μ_{PMMA}	H/m	1	
heating time	t_{heat}	s	1	
simulation time	t_{sim}	s	10	
time increment	t_{inc}	s	0.1	
density PMMA dry	$\rho_{PMMA, dry}$	kg/m³	1,130	
density PMMA wet	$\rho_{PMMA, wet}$	kg/m³	1,350	
density Co28Cr6Mo	$\rho_{Co28Cr6Mo}$	kg/m³	8,830	

Supplementary Table S8 Input parameters of the model considered as temperature-dependent. Thermal conductivities are averaged from four specimens. Specific heat capacities were average from two measurements each.

	symbol	temperature				unit
		25	50	100	150	°C
thermal conductivity PMMA dry	$\lambda_{PMMA,dry}$	0.2057	0.2063	0.2417	0.213	W/mK
thermal conductivity PMMA wet	$\lambda_{PMMA,wet}$	0.2333	0.2204	0.2257	0.2447	W/mK
thermal conductivity Co28Cr6Mo	$\lambda_{Co28Cr6Mo}$	13.11	13.81	15.05	16.15	W/mK
specific heat capacity PMMA dry	$c_{PMMA,dry}$	1,276	1,399	1,646	1,893	J/kgK
specific heat capacity PMMA wet	$c_{PMMA,wet}$	1,323	1,444	1,678	1,893	J/kgK
specific heat capacity Co28Cr6Mo	$c_{Co28Cr6Mo}$	447	462	480	495	J/kgK

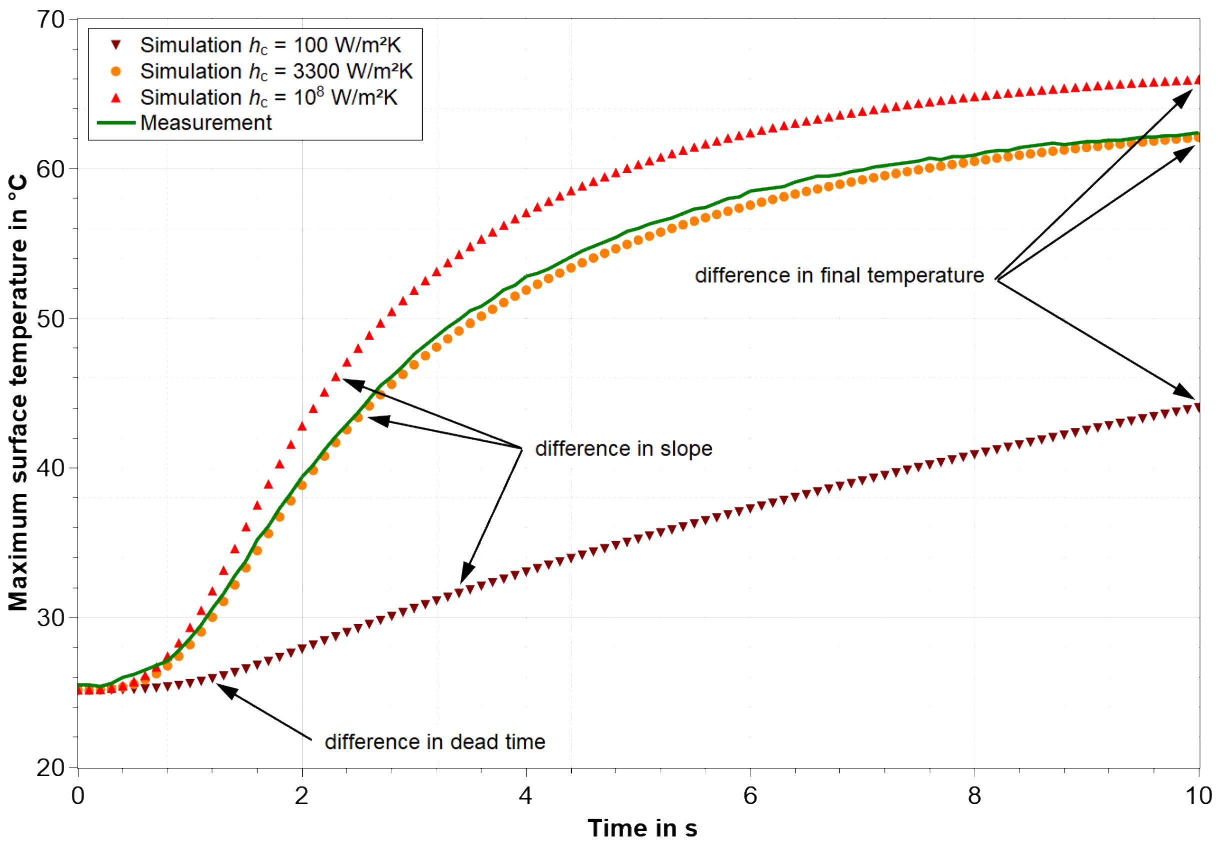

Supplementary Figure S9 Comparison of measurement and simulation data for overestimation, correct estimation and underestimation of thermal contact conductance

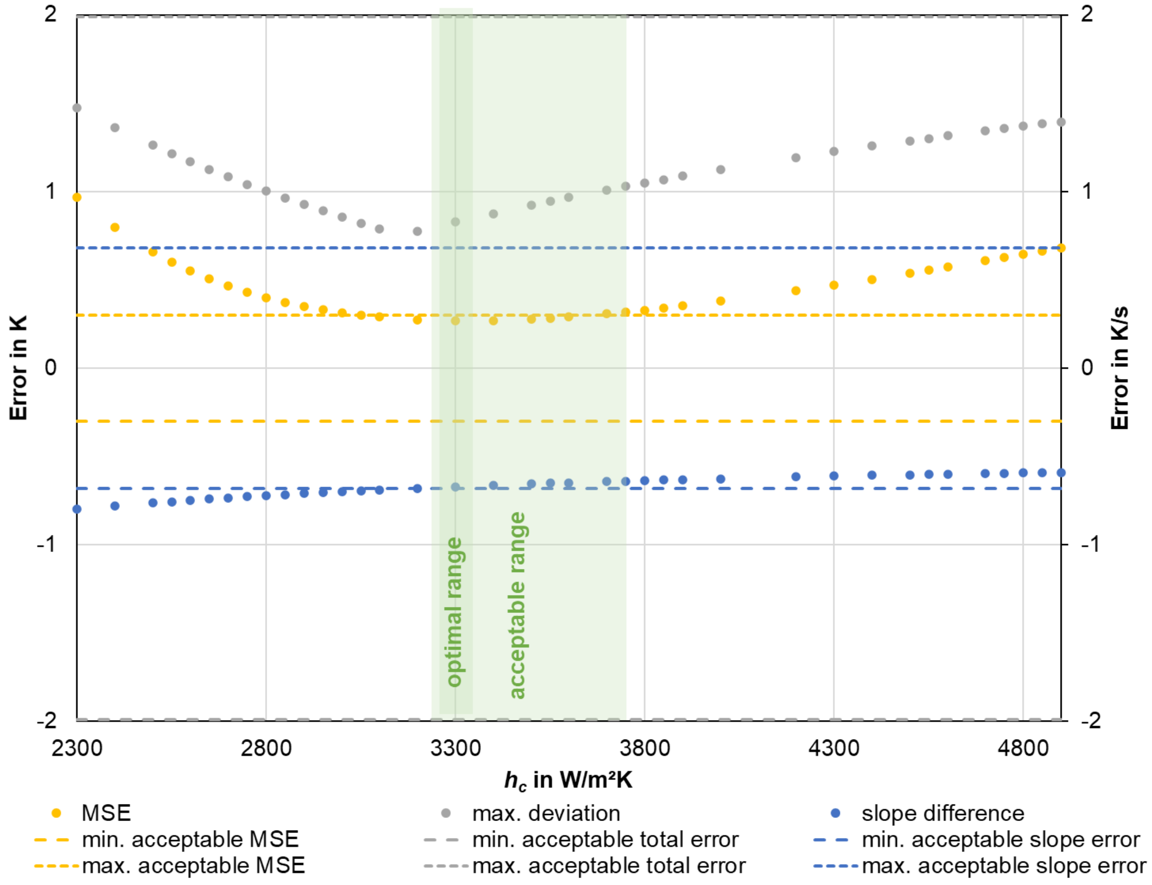

Supplementary Figure S10 Comparison of simulation quality for varying values of h_c for specimen 2 with the acceptable range in light green and the optimal range in dark green; acceptable errors are indicated by dashed horizontal lines

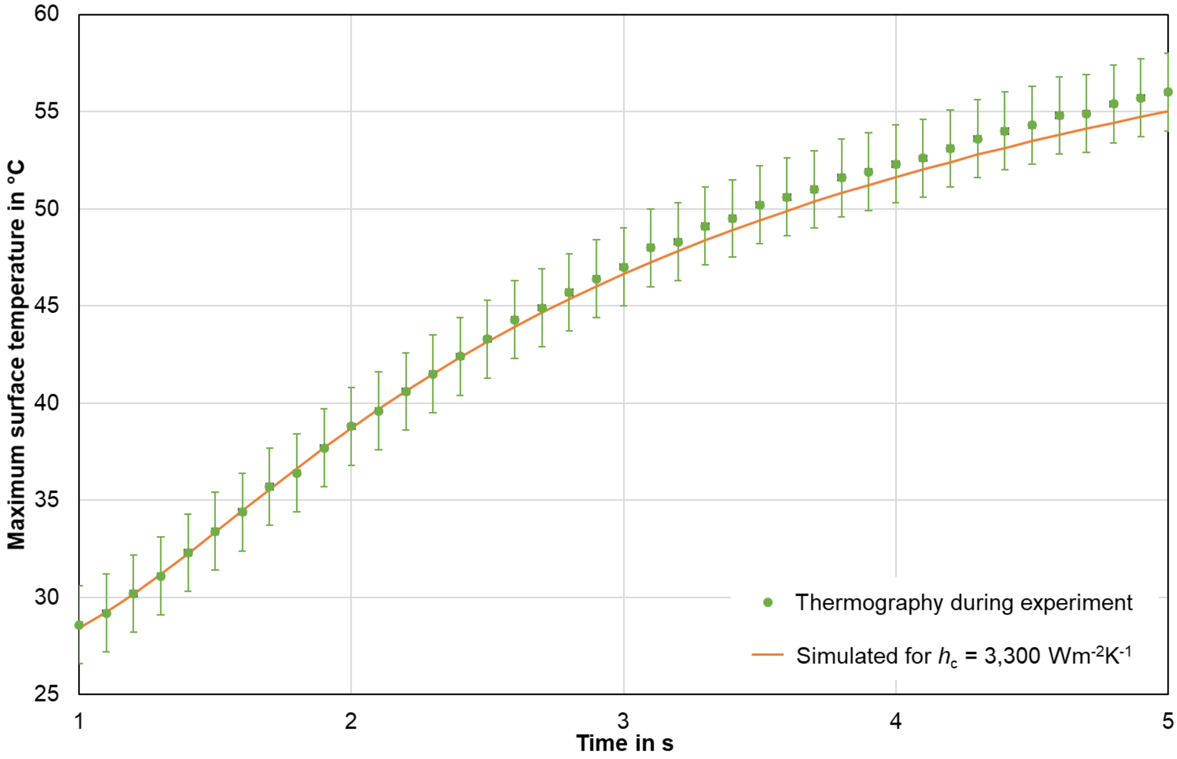

Supplementary Figure S11 Comparison of maximum surface temperature for specimen 2 as modelled with a value of h_c = 3,300 $Wm^{-2}K^{-1}$ to thermography data with error indicators of ±2 °C representing thermography accuracy

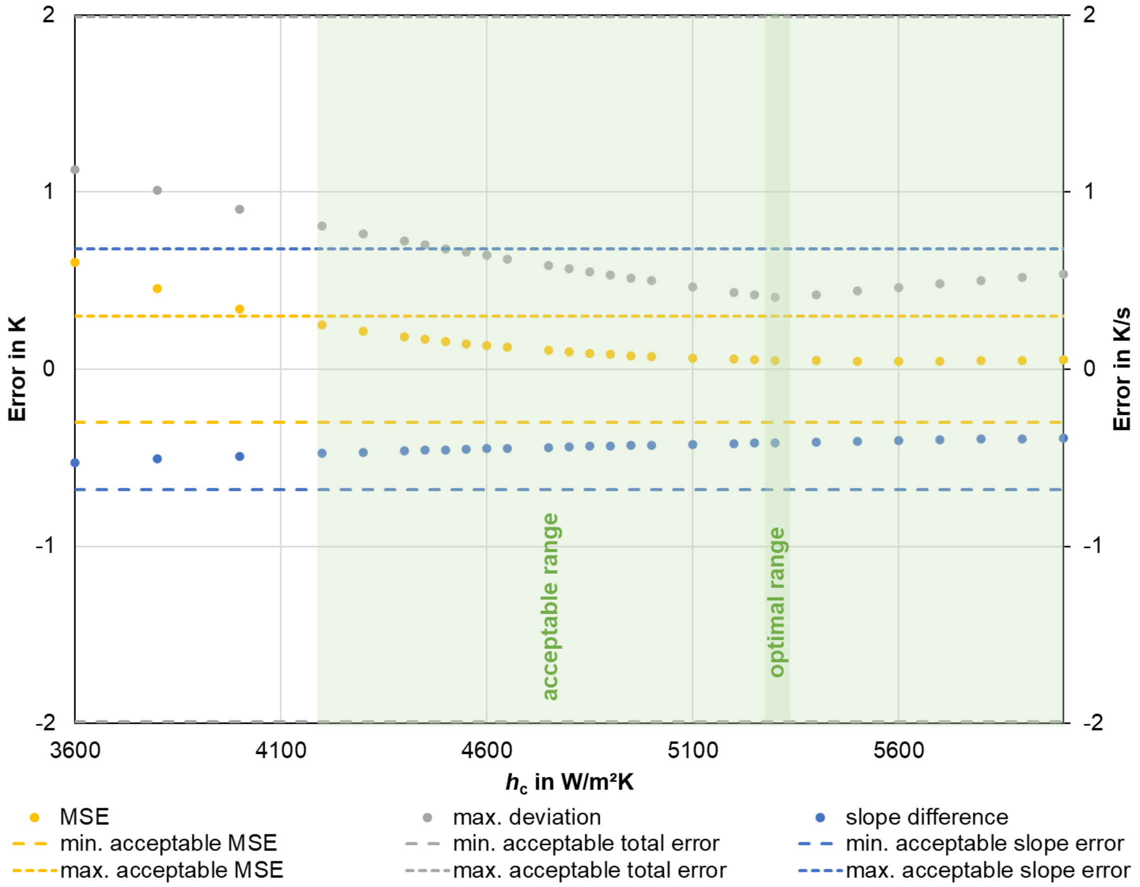

Supplementary Figure S12 Comparison of simulation quality for varying values of h_c for specimen 7 with the acceptable range in light green and the optimal range in dark green; acceptable errors are indicated by dashed horizontal lines

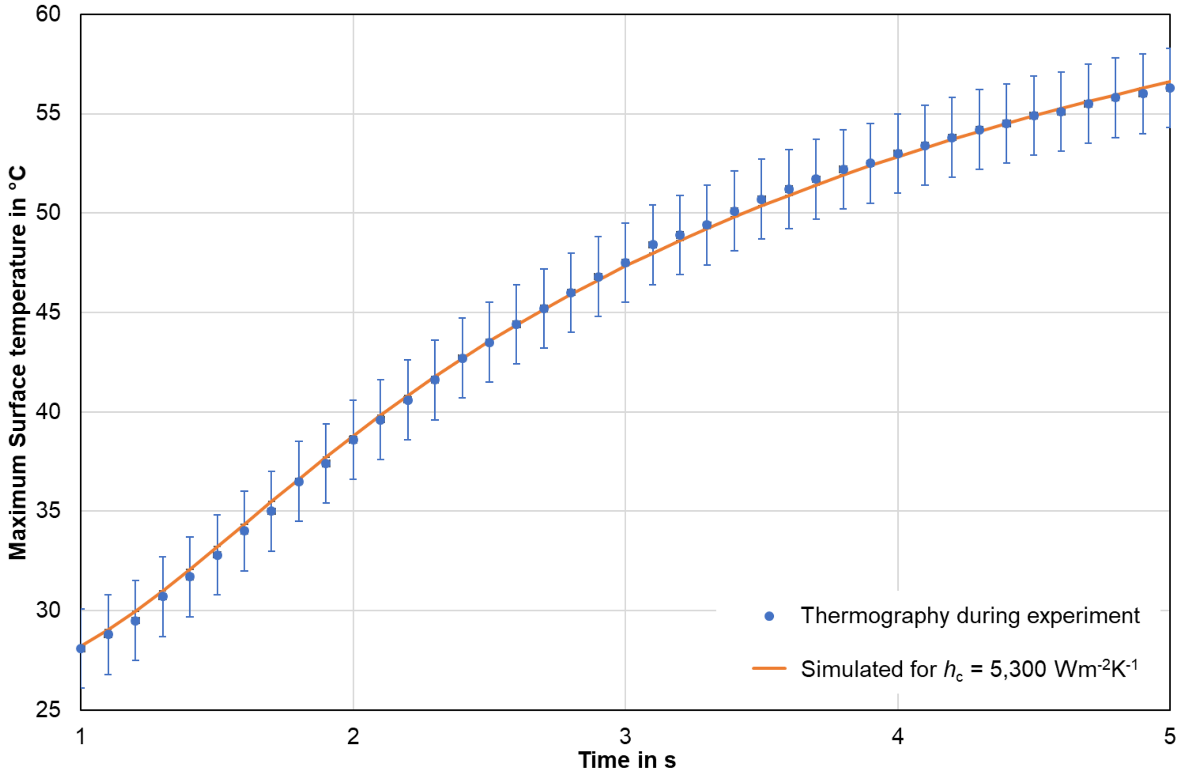

Supplementary Figure S13 Comparison of maximum surface temperature for specimen 7 as modelled with a value of h_c = 5,300 Wm^{-2}K^{-1} to thermography data with error indicators of ± 2 °C representing thermography accuracy

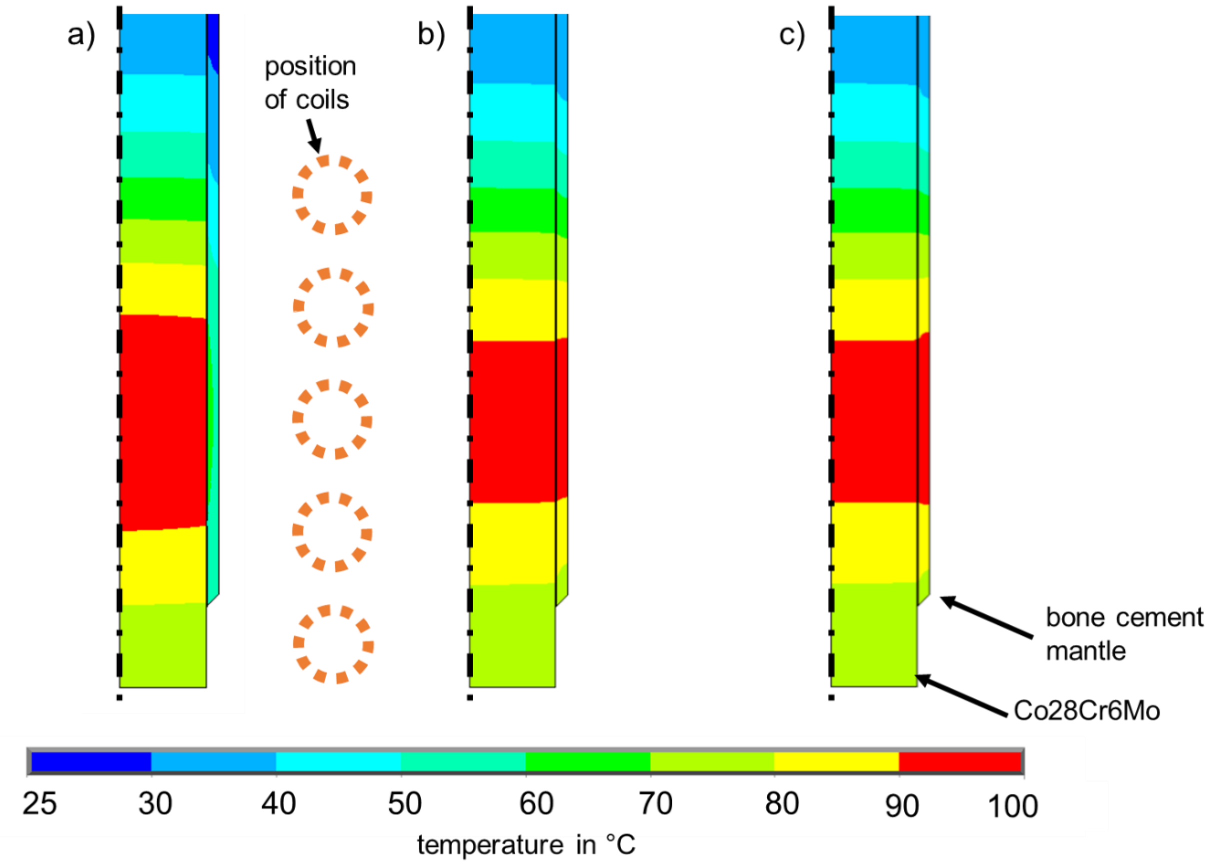

Supplementary Figure S14 Examples of resulting temperature distributions in the cross section of the specimens for a) underestimation (distribution calculated for a heating time of 1 s and a simulation time of 10 s with a value of h_c = 100 $Wm^{-2}K^{-1}$) b) suitable estimation (h_c = 3,100 $Wm^{-2}K^{-1}$) and c) overestimation of the TCC (h_c = 10^8 $Wm^{-2}K^{-1}$); the position of the individual coil windings relative to the specimen is indicated in image a)

References

1. Evers, P. *et al.* Characterization and modeling of the inductive heating of CoCrMo hip implants to facilitate intentional removal. *2024 IEEE Int. Symp. Med. Meas. Appl. MeMeA* (2024).

2. J. Powell, A. Papadaki, J. Hand, A. Hart, and D. McRobbie, “Numerical simulation of SAR induced around Co-Cr-Mo hip prostheses in situ exposed to RF fields associated with 1.5 and 3 T MRI body coils,” *Magn. Reson. Med.*, vol. 68, no. 3, pp. 960–968, 2012, doi: 10.1002/mrm.23304.

3. “UNS R30075 (ASTM F75, ISO 5832-4) Co-Cr-Mo Alloy :: MakeItFrom.com.” Accessed: Dec. 10, 2024. [Online]. Available: https://www.makeitfrom.com/material-properties/UNS-R30075-ASTM-F75-ISO-5832-4-Co-Cr-Mo-Alloy

4. “Appendix B: Material Properties,” in *Foundations for Microstrip Circuit Design*, John Wiley & Sons, Ltd, 2016, pp. 635–642. doi: 10.1002/9781118936160.app2.
